# Salmonella Control Programme of Pig Feeds Is Financially Beneficial in Finland

**DOI:** 10.3389/fvets.2019.00200

**Published:** 2019-07-09

**Authors:** Jarkko K. Niemi, Katriina Heinola, Maria Simola, Pirkko Tuominen

**Affiliations:** ^1^Bioeconomy and Environment, Natural Resources Institute Finland (Luke), Seinäjoki, Finland; ^2^Bioeconomy and Environment, Natural Resources Institute Finland (Luke), Helsinki, Finland; ^3^Risk Assessment Unit, Finnish Food Authority, Helsinki, Finland

**Keywords:** *Salmonella*, cost–benefit analysis, stochastic simulation, feed, contamination, risk, pig production

## Abstract

To promote public health, Finland has adopted a stringent *Salmonella* control policy. However, the rationale of *Salmonella* control in pig feeds has been debated after a European Union (EU)-wide cost–benefit analysis, which provided mixed, country-specific results on whether control measures are economically beneficial. The aim of this study was to analyze the costs and benefits of current pig feed *Salmonella* control in Finland compared to a reduced control scenario. In addition, this study contributes to the literature by looking at the costs across stakeholder groups. The costs of preventive and monitoring measures were assessed, and a Monte Carlo model was developed to simulate costs caused by *Salmonella* contaminations along the pork supply chain (including feed importation, commercial feed manufacturing, feed transportation, mobile feed mixers, pig farms, slaughterhouses) and because of human salmonellosis originating from contaminated feed. The data were collected from official records and feed sector operators by surveys and interviews. The prevalence of *Salmonella* was obtained from a previously conducted risk assessment study. The total costs of pig feed *Salmonella* control were estimated on average to be €4.2–5.4 million per year (95% of simulated years between €2.1 and €9.1 million) for the current control scenario, and €33.8–34.8 million per year (95% €2.2 to €26.0 million) for the reduced control scenario. In the reduced control scenario, the monitoring and prevention costs were decreased down to €1.1–2.1 million, and the costs of *Salmonella* contaminations and human salmonellosis were up by €32.7 million when compared to the current control scenario. The results suggest that the current pig feed *Salmonella* control policy of Finland is economically profitable. It can reduce the costs caused by feed-related *Salmonella* contaminations on average by €29.4 million per year and provides public health benefits. Pig feed *Salmonella* control can support the effectiveness of the Finnish *Salmonella* Control Programme. The current pig feed *Salmonella* control policy benefits the consumers, while a substantial part of the costs are covered by feed operators. In order to increase the acceptability of current policy, greater attention to the allocation of financial responsibilities regarding the control measures may be required.

## Introduction

*Salmonella* is a bacteria which can cause food-borne illness and negatively affect human health. It can be transferred between humans and nonhuman animals through contaminated food, drink, or the environment. Salmonellosis can incur economic losses to society due to increased health care costs, lost working days when people are ill, and mortality (1). Ao et al. (2) estimated that that 3.4 (range 2.1–6.5) million human cases of invasive non-typhoidal *Salmonella* disease occur globally each year. In the European Union member states, *Salmonella* is the most frequently reported pathogen in food (3). In 2013–2017, there were 19.7–21.0 human cases of salmonellosis per 100,000 population reported in the European Union (4). In Finland, ~1,500 to 3,000 salmonellosis cases (26–58 cases per 100,000 population) have been reported each year (5), and about 2 out of 3 cases have been acquired from abroad. However, 70–90% of the actual cases have been estimated to remain unreported (6, 7).

The association between *Salmonella* contamination in feed and pigs is well known and epidemic feed-borne *Salmonella* outbreaks have occasionally been experienced. For instance, in 2003, a feed-borne outbreak of *Salmonella cubana* occurred in Sweden as a result of contamination in a feed plant. *Salmonella cubana* was detected in 49 of 77 pig farms having received possibly contaminated feed (8). A study published in 2008 (9) found that the prevalence of *Salmonella* spp. in slaughter pigs within the European Union (EU) was 10.3%. The estimates ranged by member state from 0% in Finland to 29% in Spain (9). The European Food Safety Authority (EFSA) (10) has estimated that around 10–20% of human *Salmonella* infections in EU may be attributable to the pig reservoir as a whole. Their quantitative microbial risk assessment analysis suggested that an 80 or 90% reduction in lymph node prevalence should result in a comparable reduction in the number of human cases attributable to pig meat products. EFSA (10) further suggests that by feeding only *Salmonella*-free feedstuffs, a reduction of 10–20% in high-prevalence member states and 60–70% in low-prevalence member states were foreseen in slaughter pig lymph node prevalence.

The literature shows several measures which can be used to mitigate the risk of *Salmonella* [see, e.g., review by Andres and Davies (11)]. Following appropriate biosecurity and feed-based interventions are among the most important measures to be taken. Preventive measures to control diseases can also lead to increased public trust toward the food system [see Clark et al. (12)]. To promote public health, Finland has adopted a stringent *Salmonella* control policy (13). The policy aims to decrease the occurrence of *Salmonella* across the food supply chain. The Finnish *Salmonella* Control Programme (FSCP) (14) covers the *Salmonella* surveillance and risk management measures for pigs, beef, and poultry, and the meat and eggs derived from these. At a national level, it aims to protect consumers through ensuring that <1% of animals and meat are contaminated with *Salmonella*. The program takes measures to reduce the risk of *Salmonella*-positive eggs or meat from reaching the market (15).

FSCP has been regarded to achieve high food safety targets of meat, milk, and egg food chains without high costs (16). FSCP has been regarded as economically profitable in the broiler meat supply chain (17–20), whereas the financial viability of *Salmonella* control in the pig sector or for feedstuffs has not been investigated. *Salmonella* control in pig feeds has been considered only by an EU-wide cost–benefit analysis (21), which evaluated five different *Salmonella* control options across the EU member states. These options varied depending on whether biosecurity at the farm, interventions based on high or low *Salmonella* prevalence, or transport and slaughterhouse measures were taken into account. In most cases, the benefits of the control options were lower than their costs. In the case of Finland, two options were financially viable [i.e., Net Present Value (NPV) was positive]: these included an establishment of a *Salmonella* control support unit and some increased sampling, as well as feed control measures either with or without transport and slaughterhouse measures. By contrast, an establishment of a *Salmonella* control support unit and some increased sampling with or without specific feed practices and farm-level biosecurity were not financially viable (21).

Studies provide conflicting evidence on whether feed-related interventions to control *Salmonella* are economically viable. While some studies have found that feed-related interventions can be financially beneficial either as such or as part of a wider program [e.g., Lawson et al. (22); Sundström et al. (23); Gavin et al. (24), FCC consortium (21)] other studies have found their costs higher than the benefits [e.g., Miller et al. (25); Goldbach et al. (26)].

While feeds, especially imported feed materials and feeds, are considered the most important source of *Salmonella* contaminations in animals, FSCP does not cover feedstuffs. However, the feed law does not allow feed in Finland to contain *Salmonella*. The feed operators must indemnify damage for a buyer of feed when the feed does not comply with the legal requirements, even when the incompliance is not caused by intent or negligence (Finnish Feed law, 86/2008). This principle is called strict liability. The prevalence of *Salmonella* in the Finnish pork chain is low [e.g., Maijala et al. (16)], and Jensen and Unnevehr (27) suggest that intervention costs increase when the desired level of pathogen approaches zero. Hence, is it relevant to investigate whether a control program is economically viable.

To comply with the regulations, feed operators apply voluntary and mandatory prevention and monitoring measures. This, together with the FCC consortium (21) results, has stimulated discussion on whether the current *Salmonella* control in pig feeds is cost-effective. The issue is scientifically and empirically interesting from several perspectives. Firstly, the overall financial viability of the pig feed *Salmonella* control is a pertinent issue. Secondly, another relevant question is how the costs and benefits of pig feed *Salmonella* control are distributed along the supply chain. This is important because if the costs of control are paid by different stakeholders rather than those who benefit from the measures, then some stakeholders may have inadequate economic incentives to promote stringent *Salmonella* control. Hence, this article contributes to the literature by studying the costs and benefits of the pig feed *Salmonella* control program across stakeholder groups. The data were collected from official records and from feed sector operators through surveys and interviews, conducted simultaneously with a risk assessment related to Finnish pork production. The epidemiological analysis was obtained from Rönnqvist et al. (28). The analysis takes into account the current *Salmonella* control, including statutory and voluntary measures, and compares the current situation with a reduced control scenario where current statutory *Salmonella* controls are applied in pig farms but not in full for the commercial feed manufacturers or feed or feed material importers.

## Materials and Methods

### Salmonella Control Scenarios

Two scenarios to control *Salmonella* in pig feeds were compared. The first scenario corresponded to the current *Salmonella* control policy. The second scenario represented a reduced control policy. The main differences between the scenarios are represented in Table 1. In the reduced control scenario, fewer prevention and monitoring activities were targeted on imported feed, commercial feed manufacturing, feed storage, and mobile feed mixers in the reduced control than in the current control scenario. Some control measures were eliminated completely. If *Salmonella* was detected in feed, no actions were assumed to be taken to eliminate the pathogen from feed under the reduced control scenario. *Salmonella* contaminations in pigs or pork were assumed to be treated similarly in both scenarios.

**Table 1:** Measures applied in the current and in the reduced *Salmonella* control scenario, and the proportion of measures applied in the reduced control scenario (% of the current control policy costs which are incurred in the reduced control scenario)^a^.

***Salmonella* control measures applied**	**Current control^**a**^**	**Reduced control**
**CONTROL AT IMPORT OF FEED AND FEED MATERIALS**		
Official control measures	Yes	No (0%)
Self-monitoring, sampling	Yes	Reduced (50–90%)
Quarantine storage for high-risk feed	Yes	No (0%)
Eradication of *Salmonella* when detected	Yes	No (0%)
**CONTROL AT COMMERCIAL FEED MANUFACTURING**		
Treatment of feed, labor and materials^b^	Yes	Reduced (95%)
Maintaining appropriate hygiene	Yes	Yes (100%)
Pest control	Yes	Yes (100%)
Official control, sampling	Yes	No (0%)
Samples as self-control	Yes	Reduced (50–90%)
Self-monitoring and related documentation	Yes	Reduced (80%)
Eradication of *Salmonella* when detected	Yes	No (0%)
**CONTROL AT MOBILE FEED MIXERS**		
Treatment of feed	Yes	Reduced (50%)
Official control and sampling	Yes	No (0%)
Maintaining appropriate hygiene	Yes	Yes (100%)
Samples as self-control	Yes	Reduced (50–90%)
Self-monitoring and related documentation	Yes	Reduced (75%)
Eradication of *Salmonella* when detected	Yes	Reduced (0–100%)
**CONTROL AT PIG FARMS**		
Treatment of feed, acid treatment	Yes	Yes (100%)
Sampling	Yes	Yes (100%)
Maintaining appropriate hygiene	Yes	Yes (100%)
Eradication of *Salmonella* when detected	Yes	Yes, if detected in pigs (100%)
**CONTROL AT SLAUGHTERHOUSE**		
Extra measures if *Salmonella* is detected	Yes	Yes (100%)

a *In the current control policy scenario, all costs and measures were applied in full, which corresponds to 100% adoption rate in the reduced control scenario. Percentages in the reduced control scenario indicate which proportion of current control measures were applied, and hence, which proportion of costs were incurred when compared to the current control policy scenario*.

b *Heat treatment for production volumes more than 6 million kilograms, except liquid feed, vitamin, and mineral mixes*.

While the price parameters were similar for both scenarios, there were differences in the parameters representing a prevalence of *Salmonella* and differences in whether the measures to monitor, prevent, or eradicate the bacteria were taken.

### Computational Model

In order to assess the costs and benefits of pig feed *Salmonella* control in Finland, a model to simulate two types of costs for a given control scenario was developed. The costs were simulated on an annual basis. The first type of costs was costs associated with measures to prevent *Salmonella*. These included the cost of feed (heat) treatment and other measures to reduce the risk of *Salmonella* contamination, cleaning measures and pest control at different stages of the supply chain, statutory *Salmonella* sampling and official control checks made by authorities, and self-monitoring measures related to *Salmonella* control in commercial feed manufacturing. The second type of costs includes costs caused by realized *Salmonella* outbreaks and *Salmonella* arising from the contamination of pig feed. These costs included the costs of cleaning facilities where *Salmonella*-contaminated feed or pigs had been detected; the costs of treating contaminated feed with substances permitted to treat feed; losses due to idle production capacity at feed manufacturing, feed storage, or farms; costs due to *Salmonella* observed at slaughterhouse or in pork; costs due to human cases of salmonellosis; and tracing, product recalls, and labor and material input associated with these measures. The model considered these two types of costs to different stakeholders across the pork supply chain. The stakeholders included feed importers, commercial feed manufacturers, feed transporters, mobile feed mixers, pig farms, slaughterhouses, taxpayers (government), and consumers of pork. The benefits of effective *Salmonella* control were because of a reduced costs burden caused by realized *Salmonella* outbreaks and *Salmonella* arising from the contamination of pig feed (possibly leading to contamination of pigs or their environment).

The total annual costs (*L*) of control and prevention measures plus costs caused by *Salmonella* contaminations and human infections in each scenario were calculated as:

$$\begin{array}{l} L=\sum_{h=1}^{H}P_{h}C_{h}+\sum_{i=1}^{24}\;{\text{pa}\prime }_{i}w_{i}Q_{i}+\sum_{j=1}^{9}\;{\text{pa}\prime \prime }_{j}w_{j}Q_{j}\\ \;+\;\sum_{k=1}^{14}\;{\text{Pft}}_{k}w_{k}Q_{k}d_{k}\;+\sum_{r=1}^{2}\;{\text{Papp}}_{r}w_{r}Q_{r}\theta_{1,r}\\ \;+\sum_{m=1}^{4}\;P_{m}w_{m}Q_{m}\theta_{m}fd_{m} \end{array}$$

where *h* refers to one of *H* cost items associated with preventive or monitoring costs; C_*h*_ refers to the total costs of item *h*, which is implemented fully or partly because of the goal of reducing *Salmonella* contamination; P_*h*_ is the proportion of prevention or monitoring measures' costs C_*h*_ that are associated with item *h* (i.e., if a measure is adopted for multiple reasons, this parameter indicates the contribution of the *Salmonella* control to the costs); *i, j, k*, and *r* are indices representing feed material (*i*), feed (*j, k*), or animal type (*r*), respectively, in connection with measures associated with the treatment of *Salmonella*-contaminated materials, animals, or humans; *m* refers to “severity” of human salmonellosis; pa′_*i*_ pa″_*j*_, Pft_*k*_, Papp_*r*_, and P_*m*_ represent the probability of *Salmonella* contamination or prevalence of *Salmonella* contamination occurring in *i, j, k, r*, or *m*; *w* is the cost caused by *Salmonella* contamination, or eradication of the pathogen, in *i, j, r, k*, or *m*; d_*k*_ is the proportion of true infections that will be detected; *d*_*m*_ is the proportion of each type of human infection; θ is the proportion of infections related to contamination in feed; and *Q* represents the quantity of pig feed materials, pig feed, pigs, or humans in the study population.

For feed materials at import and storage prior to feed manufacturing, pa′_*i*_ is the apparent prevalence (pa′) of *Salmonella* in feed material batches of 25 tons. For costs incurred at the industrial feed manufacturing stage, the apparent prevalence of *Salmonella* in manufactured compound or complete feed (pa″_*j*_) was used. The occurrence of *Salmonella* contamination in pigs at the farm was modeled by using the probability of contamination (Pft_*k*_), and the probabilities were specific to feeding type and animal (complete feeds for sows, piglets or fattening pigs, farm-made feeds plus complementary feeds for sows, piglets or fattening pigs). As this was the true prevalence, only the proportion d_*k*_ was considered to result in costly eradication measures.

The incidence of *Salmonella* in slaughter pigs was assigned with the observed prevalence of infections (Papp) represented by the prevalence in the lymph node samples, and the costs for infection (w_*r*_) were relative to the number of pigs that were assumed to be influenced in the batch when a *Salmonella*-positive pig was detected. Finally, the annual prevalence of *Salmonella* infections in humans P_*m*_ was determined as a proportion of observed infections that could, according to the source attribution model, be linked to pig feeds. P_*m*_w_*m*_Q_*m*_θ_*m*_*f* *d*_*m*_ therefore represent the product of the prevalence in humans, size of the population in Finland, reporting factor *f* = 11.5 (21), proportion of infections associated with contamination in feed, and the proportion of infections associated with each type of human infection and cost w_*m*_ per infected person. The following sections characterize information used to parameterize the model.

### Data

The information needed for the cost–benefit analysis was gathered from several sources, including the reports of the former Finnish Food Safety Authority (Evira), which has been called the Finnish Food Authority since January 1, 2019, the Finnish Farm Registry, and from a questionnaire that administered to the feed producers in Finland. The data were collected, and the simulations were carried out for 2013.

Data on the volume of pig meat and feed production, and the number of pig farms and pigs in Finland were based on the statistics of Luke (29) and Evira (30). The costs of preventive measures were defined for seven large commercial feed manufacturers, which produced in total 290,000 tons of commercial pig feed in 2013, as well as for 12 mobile feed mixers, which produced 33,000 tons of pig feed, and all the pig farms in Finland (~1,600 farms in 2013). Commercial pig feed producers use the vast majority of the imported high-risk feed material listed in the Decree of the Ministry of Agriculture and Forestry on the pursuit of activities in the animal feed sector 548/2012. For this reason, the amount and cost of imported-high risk feed were estimated for these operators' production. The costs related to the time used for self-monitoring were assessed for pig farms that were registered as feed manufacturers (about 400 farms), as only these farms have a statutory requirement for self-monitoring.

The questionnaire was sent to nine feed mill operators, of which six responded. From the 432 pig farms that had reported feed manufacturing, 61 returned completed questionnaires and 53 other farms informed that they no longer had pork production. Only 2 mobile mixers out of 12 filled in the questionnaires. All the respondents did not answer all of the questions. Therefore, any missing operator information was added by using information sourced from other similar operators. Besides the questionnaire, complementary data were gathered by interviewing a mobile mixer and the staff of a feed mill. In addition to the survey and the interviews, cost and price information was collected from laboratories; service providers collaborating with the feed industry, such as pest control operators and warehouse operators; and experts in the feed industry.

### Costs of Measures to Prevent *Salmonella*

Costs of preventive measures related to the import of feed and feed materials are caused by statutory *Salmonella* sampling (self-monitoring and official monitoring), possible fees by authorities, and quarantine storage for high-risk feed materials. The Finnish Food Authority (formerly Evira) is responsible for controlling *Salmonella* in feed. The control is based on legislation and described in the annual control plans. This covers the control of feed mills and other operators. The authorities carry out spot checks for the imported and non-imported feeds and control that feeds meet legal requirements.

In Finland, *Salmonella* samples are taken as official sampling from high-risk feed imported from outside the EU member states, and mainly as self-monitoring from the high-risk feed within the EU member states. More intensive monitoring is currently required for high-risk than non-high-risk feed materials. A Ministry of Agriculture and Forestry decree on the pursuit of activities in the animal feed sector (548/2012) states that high-risk feed materials listed in annex 3 of the decree (for example, soybean, rapeseed, and meals derived from these are considered as high-risk materials) and feed imported from outside the EU member states must be analyzed by official sampling, and by self-monitoring when importing from the member states. The amounts of imported high-risk feed materials used for pig feeds were evaluated based on the amount of manufactured pig feed per operator and the sample pig feed recipes obtained from feeding experts. The imported high-risk feed material attributed to pig feed production was about 55,000 tons in 2013. The additional warehouse capacity for the prolonged storing of feed at the harbor because of waiting for laboratory results for 4–6 days was included in the costing.

For the commercial feed manufacturers, such as feed mills, costs related to preventive measures consisted of *Salmonella* sampling, the treatment of feed, maintaining appropriate hygiene and cleanliness in the facility, self-monitoring, pest control, and official inspections, including the official *Salmonella* samples. Feed manufacturing was controlled by inspections and sampling based on the authorities' control plan, which focuses on those control points considered most risky. All the feed manufacturers were required to have a self-control system for a hazard analysis and critical control points (HACCP). Sampling and other measures are defined by the HACCP. Large feed mills were inspected annually. It was mandatory for all feed operators who produced more than 6 million kilograms of feed per year to treat their products with heat or acid to mitigate *Salmonella* in feed (Finnish feed law 86/2008, 23 §). Based on the survey, only two of the operators used other treatments than heat treatment.

Acid treatment, maintaining appropriate hygiene related to mobiles, self-monitoring measures, and official inspections were considered as *Salmonella* control measures applied by mobile feed mixers. In the case of pig farms, costs included in the assessment were *Salmonella* sampling, pest control, maintaining appropriate hygiene and cleanliness in feed storages and feeding facilities, and acid treatment when using liquid feeding (about 70% of farms).

The costs were assessed by first estimating the total annual cost of monitoring and preventive control measures. These costs were obtained from the stakeholder survey and interviews. Second, a share of these costs was attributed to pig feed in relation to the share of manufactured pig feed of all feed, since especially commercial feed production operators purchase materials and produce feed for many types of animals, and further to *Salmonella* control, as all the measures besides *Salmonella* sampling were assessed to be carried out also to prevent other diseases than *Salmonella*. The share attributed to *Salmonella* was evaluated by the feed sector experts individually to each measure, and they represent the proportion of costs that could be saved if *Salmonella* control were to be discontinued. This approach was chosen in order to take into account that preventive measures may mitigate both *Salmonella* and other diseases. As feed heat treatment would be carried out to some extent also when there was no mandatory *Salmonella* control, zero percent of the costs of treatment equipment maintenance and installation costs, and 20% (feed mills), 50% (mobile mixers), and 10% (farms) of the labor and material costs were assigned to *Salmonella*. Overall, 25% of the costs of official feed control, maintenance of appropriate hygiene and cleanliness, pest control (except 14% for feed mills), and self-monitoring (except 50% for feed mills) were assumed to be attributed to *Salmonella*. Potential impacts of preventive costs to market-clearing prices and quantity traded were not considered in the current analysis because the costs were fairly small when compared to the volume of Finnish pork production.

The parameters assumed to represent the costs of preventive measures in the current control scenario are provided in Table 2. Table 1 summarizes the proportion of preventive and control costs incurred in the reduced *Salmonella* control scenario when compared to the current control policy scenario.

**Table 2:** Parameter values assumed to represent the costs (€1,000 per year) of preventive measures in the current *Salmonella* control scenario per different stakeholder group.

**Cost item**	**Feed importation**	**Feed mills**	**Mobile mixers**	**Pig farms**	**Source or method of data extraction**
Sampling as self-monitoring	42	150	1.8	44	Survey, expert interview
Hygiene and cleaning		24	0.9	364–756	Survey
Pest control		1		28	Expert interview
Time used for other self-control measures		9	0.3–1.0	9–10	Survey
Feed treatments		974–1,499	1.0	67–329	Survey, Wierup and Widell (31)
Official control and sampling	56	17–18	2.8–3.4		Expert interview, national monitoring data
Prolonged storage	7–11				Expert interview
Total	105–109	1,174–1,701	5–6	512–1,166	

*Empty items in the table were assumed zero*.

### Cost of Human Salmonellosis and *Salmonella* Contaminations in the Pork Chain

As a consequence of the *Salmonella* control, fewer *Salmonella* contaminations along the pork supply chain and fewer human cases of salmonellosis can occur, thus reducing the costs of illness and contaminations along the food supply chain. The costs related to *Salmonella* were first estimated per *Salmonella* case, and then the number of cases was simulated for the two control scenarios by using a seeded Monte Carlo simulation model programed in MATLAB R2014b 8.4.0.150421 (MathWorks Inc., USA) to calculate the total annual costs of *Salmonella* contaminations and infections.

Costs caused by *Salmonella* contaminations included the statutory measures and additional voluntary measures taken by feed importers, feed mills, mobile feed mixers, pig farms, and slaughterhouses in accordance with the current control policy to eliminate *Salmonella* when it was simulated to occur in the pork supply chain. Measures included additional samples taken to verify *Salmonella* contamination and freedom from the bacterium; washing, cleaning, and disinfecting of facilities contaminated with *Salmonella*; and the treatment of contaminated feed. *Salmonella*-positive findings had to be communicated to authorities who are in charge of further actions carried out in cooperation with the feed business operator. If a sample from a feed consignment imported from the EU or from outside the EU was found positive for *Salmonella*, the contaminated feed was assumed to be treated with heat or acid. In the event of *Salmonella* contamination in pigs or pig farms, the cost included the treatment or culling and rendering of infected animals, business interruptions caused by restrictive measures which prevent the farm from selling animals, labor effort by authorities and stakeholders to handle the case of *Salmonella* contamination, and costs caused by infections in humans (lost working time, visits to the doctor, hospitalization, mortality, and secondary diseases).

The economic burden of *Salmonella* and its sequela in humans was estimated by using the disability adjusted life year (DALY) method. DALY is a non-monetary approach to estimate health implications of a disease. The average cost per human infection was estimated to range from €530 to €620 per case. This includes the cost of health care and productivity loss of acute symptoms (gastroenteritis with a raised body temperature and bloody diarrhea), sequela (reactive arthritis, inflammatory bowel disease, and irritable bowel syndrome), and death. Death was valued at €55,000 per lost human life year (32). It was evaluated that the true number of *Salmonella* infections would be 11.5-fold (21) compared to the annual number of reported cases and that although a productivity loss can occur due to absence from work, 80% of cases would not require any acute health care as they were asymptomatic. Symptoms requiring health care were divided further into different severities, ranging from the visit to a general practitioner to the hospitalization of the patient.

Table 3 illustrates the cost parameters used to simulate the costs of *Salmonella* contaminations. All parameters (mean, median, standard deviation (SD), percentiles) describing the prevalence of *Salmonella* at different stages of the supply chain were obtained from Rönnqvist et al. (37) [further described in Välttilä et al. (38)]. Parameters for the prevalence of *Salmonella* in pig feed, feed materials, and feed-related facilities are described in Appendices 1 and 2 for the current control scenario.

**Table 3:** Parameter values assumed to represent the costs [mean cost (SD in parentheses) or range of variation] of a *Salmonella* contamination, the costs of human cases, and the costs of measures required to eradicate *Salmonella* from feed materials, feed manufacturing, feed storage, or feed processing facility; from a pig farm; or from a slaughterhouse.

**Variable**	**Parameter value**	**Source or method of data extraction**
**IMPORTATION**		
Treatment, € per lot	46,849 (SD 2,237)	Expert interview
Extra samples, € per batch	3,938 (SD 2,809)	Expert interview, laboratory
Extra rent of warehouse, € per batch	719 (SD 343)	Expert interview
**FEED MANUFACTURING**		
Disinfection and cleaning of the mill environment, € per case	1,000–1,500	Survey
Disinfection and cleaning of feed manufacturing line, production interruption for a week, additional work and compensations paid to the customers, € per case	167,500–390,000	Survey
**PIG FARMS**		
Cleaning and disinfecting a piggery, € per sow	160–431	Expert interview
Cleaning and disinfecting a piggery, € per fattening pig	106–190	Expert interview
Culling and rendering, € per farm	1,640	Authors' calculations
Culling and rendering in addition to farm, € per sow	49.50	Lyytikäinen et al. (33)
Culling and rendering in addition to farm, € per fattening pig	16	Lyytikäinen et al. (33)
Value of rendered feed, € per sow	10.35	Heinola et al. (34)
Value of rendered feed, € per fattening pig	4.04	Heinola et al. (34)
Official inspections, sampling, and self-monitoring, € per sow	139	Authors' calculations based on the feed law
Official inspections, sampling, and self-monitoring, € per fattening pig	62	Authors' calculations based on the feed law
Restricted measures, duration in days	21–259	Evira
Lost value of culled animals and costs due to business interruptions, € per fattening pig	102 + 0.41*duration of restrictive measures	Niemi et al. (35)
Lost value of animals and costs due to business interruptions, € per fattening pig (pigs not culled)	12.7 + 0.47*duration of restrictive measures	Estimated with a model similar to Niemi et al. (36)
Lost value of animals and costs due to business interruptions, € per sow (pigs culled)	665 + 0.53*duration of restrictive measures	Estimated with a model similar to Niemi et al. (36)
Lost value of animals and costs due to business interruptions, € per sow (pigs not culled)	0.6 + 1.3*duration of restrictive measures + 4.8*10^5*^duration of restrictive measures	Estimated with a model similar to Niemi et al. (36)
**SLAUGHTERHOUSE**		
Extra samples, cleaning of slaughterhouse, € per case	1,070–14,620	Expert interview
**HUMAN CONTAMINATION**		
Health care, sequela (IBS, Rea, IBD), € per case on average	530–620	Authors' calculations
Feed-borne salmonellosis, annual loss of DALYs because of acute symptoms in Finland	0.04	Authors' calculations
Feed-borne salmonellosis, annual loss of DALYs because of fatality in Finland	0.97	Authors' calculations
Feed-borne salmonellosis, annual loss of DALYs because of sequela in Finland	1.43–3.64	Authors' calculations

### Differences Between Current and Reduced Control Scenarios

Under the reduced control scenario, fewer measures were taken to prevent *Salmonella* from occurring in feed. In the event that *Salmonella* was simulated to occur in feed or feed handling facilities, no action to eradicate *Salmonella* was assumed to be taken. Hence, parameter values for the prevalence of *Salmonella* in feed materials and feeds were not of importance in the reduced control scenario when considering the cost implications of prevalence on eradication measures applicable to feed. By contrast, *Salmonella* contaminations observed in pigs or pig farms, pork, or slaughterhouses were expected to lead to the same measures being taken in both current and reduced control scenarios.

Parameters, which represent the prevalence of *Salmonella* in pigs (i.e., Papp), the probability for infection due to feed (Pft_*k*_), and the annual prevalence of *Salmonella* infections in humans (P_*m*_), were the most important parameters differing between the scenarios, because in the reduced control scenario, *Salmonella* contamination of feed was not considered to lead to an action. Under the current control policy scenario, the apparent prevalence of *Salmonella* in pigs was assumed to be presented by a parameter value of 0.139% (SD 0.061), when feed was given to sows, and by a parameter value of 0.074% (SD 0.030), when feed was given to fattening pigs. The number of human cases was simulated by using three distributions. It was assumed that of 337 reported cases in 2013, multiplied by an underreporting factor of 11.5, 12.29% (SD 3.756) were associated with pig meat, and of cases associated with fattening pigs, 33.63% (SD 14.62) were associated with pig feed. Under the reduced control scenario, the number of cases in humans and pigs was simulated to be 55.42 (SD 31.54) times the number of cases simulated under the current control policy scenario.

### Sensitivity Analysis

Previous sections described the baseline parameter values used to simulate the costs of *Salmonella* control, *Salmonella* contaminations, and human cases associated with the current pig feed *Salmonella* control, as well as with a reduced control policy scenario. In addition to these, several sensitivity analysis scenarios were simulated. In the sensitivity analysis scenarios reported in the Results section, the costs parameters for the prevention and monitoring measures were doubled (i.e., increased twofold *ceteris paribus*), compared to the baseline scenario or the cost parameters for measures taken when *Salmonella* has been detected (contaminations or human cases), which were halved (i.e., decreased 0.5-fold *ceteris paribus*). The parameters were adjusted (either increased or decreased) separately for feed importation, feed manufacturing, pig farms and the slaughtering phase, and human cases (one stakeholder group at a time); for all prevention and monitoring measures simultaneously; and for all contaminations and human cases simultaneously.

## Results

### Simulated Baseline Costs

In the current pig feed *Salmonella* control policy scenario, the total costs of measures to monitor and prevent *Salmonella* in pig feeds were €1.8–3.0 million per year (Table 4), depending on whether a high or a low estimate for the cost of control measures was used to appreciate the current control program. Costs related to the importation, commercial feed manufacturing, and farms were €0.1 million, €1.2–1.7 million, and €0.5–1.2 million per year, respectively. Under the current control scenario, simulated costs of *Salmonella* contamination at feed import process were on average €1.8 million. *Salmonella* contamination of feed origin at pig farms resulted annually, on average, in €0.4 million and at slaughterhouses in €0.1 million in losses. The costs of human salmonellosis of feed origin were simulated on average at €0.1 million per year for the current control policy. The average total cost of *Salmonella* contamination under the current control policy scenario was therefore €2.4 million, varying in 95% of simulations within the range of €0.3–6.1 million. Hence, the total costs of preventive and monitoring measures plus *Salmonella* contaminations were €4.2–5.4 million per year, with 95% of simulated years falling between €2.1 and €9.1 million.

**Table 4:** Simulated cost of *Salmonella* prevention and monitoring and *Salmonella* contaminated pig feed, pigs, and human infections (€ million per year, 95% range of variation within brackets).

	**Current control scenario**			**Reduced control scenario**		
	**Low cost control**	**High cost control**	**[CI 95%]**	**Low cost control**	**High cost control**	**[CI 95%]**
**PREVENTION AND MONITORING**						
Measures at import and storage	0.1	0.1		0	0	
Measures at feed manufacturing^a^	1.2	1.7		0.6	0.9	
Measures at pig farms	0.5	1.2		0.5	1.2	
Subtotal	1.8	3		1.1	2.1	
**COSTS CAUSED BY CONTAMINATIONS**						
Contamination at import or storage	1.8	1.8	[0.0, 4.5]	0.0	0.0	[0.0, 0.0]
Contamination at feed factory	0.0	0.0	[0.0, 0.1]	0.0	0.0	[0.0, 0.0]
Contamination at farm^b^	0.4	0.4	[0.1, 1.1]	20.5	20.5	[0.6, 80.7]
Costs to slaughterhouse	0.1	0.1	[0.0, 0.4]	6.0	6.0	[0.2, 26.1]
Costs of human infections^c^	0.1	0.1	[0.0, 0.3]	6.2	6.2	[0.1, 22.0]
Subtotal^c^	2.4	2.4	[0.3, 6.1]	32.7	32.7	[1.1, 123.9]
Total costs	4.2	5.4		33.8	34.8	

a *Includes the costs of mobile mixers*.

b *Salmonella detected in the pigs or their environment*.

c *The costs of human cases were simulated assuming a fixed average cost per case*.

In the reduced control scenario, the monitoring and prevention costs were decreased down to €1.1–2.1 million. Hence, the potential savings were only €0.7–0.9 million when compared to the current *Salmonella* control policy scenario. The lower level of control resulted in fewer preventive measures being applied, which decreased the costs, but likely increased the possibility of *Salmonella* to occur, as seen in the prevalence parameters originating from the risk assessment study (28). This increased the total costs of *Salmonella* contaminations, which were on average €32.7 million. This estimate included the costs of human cases. The contamination costs estimated for pig farms were on average €20.5 million, but the costs incurred at slaughterhouses were partially related to measures taken because of contaminations at farms. The increase in the costs of human salmonellosis was on average €6.2 million, which was more than the costs saved because of reduced preventive and monitoring measures. The total costs of reduced control scenario were estimated on average to be €33.8–€34.8 million per year.

The current control policy provided on average €29.4 million in annual benefits when compared to the reduced control scenario. The additional prevention and monitoring costs of the current control policy were estimated to be within the range of €0.7 to €0.9 million per year, whereas saved costs of contaminations under the current control policy were on average €30.3 million when compared to the reduced control scenario (in 95% simulations between €0.8 and €117.8 million).

**Figure 1:**
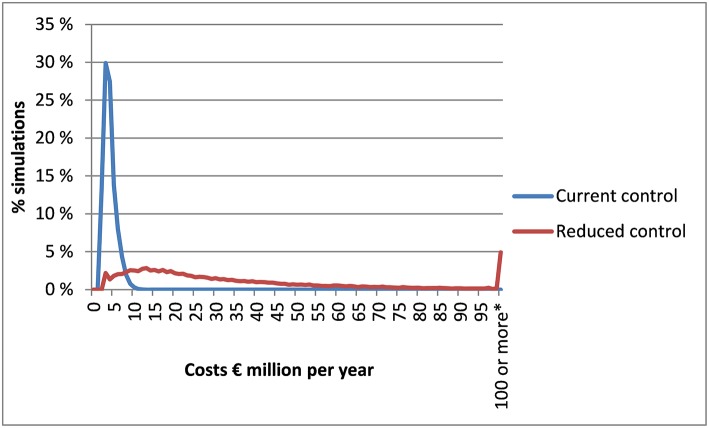
Distribution of costs simulated for measures of prevention, monitoring, and eradication of *Salmonella* in the pig feed chain, and human cases originating from *Salmonella* in the pig feed chain. ^*^Simulations exceeding €100 million per year are aggregated in this category.

The results showed a substantially larger variation in the costs of the reduced control scenario than the current control scenario (Figure 1; Table 4). The average estimates were elevated in individual years when larger outbreaks were experienced. Hence, the simulated costs of *Salmonella* control were substantially lower in the current control policy scenario than in the reduced control scenario. In the current analysis, the costs of human cases were simulated assuming a fixed average cost per case. Separating the costs by different types of human infections would have further increased the variation of simulated costs as fatality cases carried a high cost.

### Sensitivity Analysis

**Figure 2:**
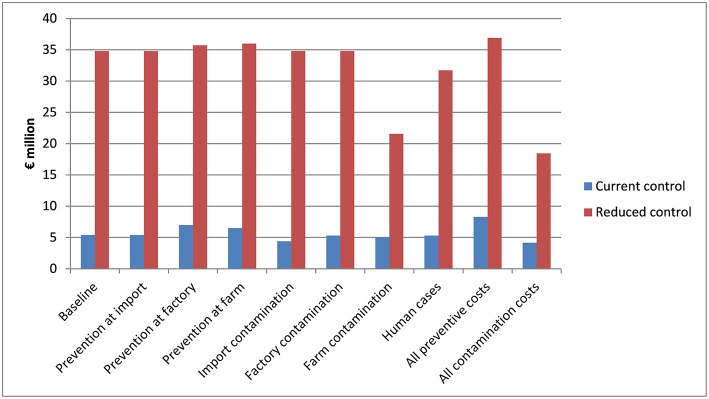
The average total costs of pig feed *Salmonella* control simulated for the baseline parameter values of “current control” and “reduced control” policy scenarios, as well as for both policies, so that the costs parameters for prevention and monitoring measures were doubled (“Prevention…”) when compared to the baseline scenario or the cost parameters for contaminations or human cases, which were halved (“Contamination…,” “Human cases”) either for one stakeholder group at a time or for all groups simultaneously.

Figure 2 reports the results for sensitivity analyses where the cost parameters of measures to prevent and monitor *Salmonella* were doubled, or the cost parameters of measures taken when *Salmonella* has been detected were halved. As Figure 2 illustrates, the costs of the current control policy were on average < €8.5 million in all sensitivity analysis scenarios representing the current control policy, whereas the costs simulated for the reduced control were on average between €21.5 and €37 million in all sensitivity scenarios. Therefore, the current control policy was financially preferred over the reduced control policy in the scenarios which were analyzed.

The results were the most sensitive to costs associated with a contamination in pigs (at farms or at slaughterhouses) and feed on pig farm. This can be anticipated because in the reduced control scenario, these costs represented approximately three-fourths of the total costs. Changes in the modeling assumptions regarding human cases of *Salmonella* also had a potentially substantial impact of the total costs. Adjusting assumptions regarding the cost parameters of prevention and control measures before *Salmonella* had occurred had a fairly small impact on the total costs.

Further analyses indicated that the costs associated with measures taken upon *Salmonella* contamination or associated with human cases had to decrease by at least 90% (*ceteris paribus*) before the costs of reduced control policy would have been, on average, at the same level, as costs simulated for the current control policy scenario. However, even then, the reduced control policy was simulated to show a larger variation of costs when compared to the current control policy. This was because the costs of contaminations and human cases varied more than the costs of prevention and monitoring measures.

## Discussion

### Economic Rationale of *Salmonella* Control in Pig Feed

In this study, the costs and benefits of current *Salmonella* control policy concerning pig feeds were assessed and compared to a reduced control scenario. The results suggested that the current pig feed *Salmonella* control policy is financially profitable as it reduced costs caused by *Salmonella* contaminations in pigs, in their environment, or in pork and human infections. Currently *Salmonella* is controlled already early in the pork supply chain. While rather small amount of costs could be saved by relaxing the current prevention and monitoring measures, simultaneously much larger additional costs would be incurred through increased human infection costs and costs caused by eradicating *Salmonella* from pigs, pig farms, and slaughterhouses. Therefore, pig feed *Salmonella* control provides both public health benefits to society and supports FSCP in it goals. The results extend earlier research results on that that FSCP is economically viable in the poultry sector [e.g., Kangas et al. (19)] and that FSCP achieves high food safety targets of meat, milk, and egg food chains without high costs (16).

Our results are in line with those of the FCC consortium (21) report in the sense that a control option with an increased sampling as well as feed control measures either with transport and slaughterhouse measures was financially profitable in both studies for Finland, and in the case of the FCC consortium (21) also for some other countries. However, our study did not look specifically for other measures than those related to feed control. In Sweden, Sundström et al. (23) found that it was not cost-effective to introduce reduced *Salmonella* control strategies. Lawson et al. (22) indicated in their comparison that using home-mixed or acidified feeds for Danish pigs was financially beneficial in some cases. Gavin et al. (24) found for the United Kingdom that two interventions using wet feed and organic acids in feed were able to provide a financial net benefit to the farms as means to control for *Salmonella*. By contrast, Goldbach and Alban (26) found that using specific home-mixed feed in herds with slaughter pigs and using acidified feed for slaughter pigs as means to control for *Salmonella* in Denmark were not socioeconomically profitable. Comparing these results and studies therefore suggests that an intervention to control *Salmonella* is not financially profitable *per se* because the profitability is influenced by context-specific factors.

The benefits of controlling *Salmonella* in pig feeds likely depends on how much feeds contribute to the risk of *Salmonella* and what is the prevalence of *Salmonella* in the country. As long as the prevalence of *Salmonella* is at the current low level, it makes sense to try to maintain the current situation in Finland and eliminate emerging cases as this can be done with a reasonably low level of costs. The results of Jensen and Unnevehr (27) suggest that intervention costs increase when the desired level of pathogen approaches zero. Miller et al. (25) suggested that changes in *Salmonella* status during processing are more important for human health risk and have a higher benefit–cost ratio when compared with on-farm strategies for *Salmonella* control. They noticed that in the contexts of the United States, benefit–cost ratios were less than unity for the on-farm strategy of meal feeding. This does not comply with our results.

Reducing feed-related *Salmonella* control measures from the current level, the risk of *Salmonella* prevalence could rise and this would have negative economic consequences to society. In the alternative situation, the number of feed-borne human salmonellosis cases would increase, which would have a negative effect on the health care cost, but also the productivity of labor due to more absences from work. Approximately €6.2 million were saved because of improved public health (i.e., human cases) under the current *Salmonella* control policy. Therefore, the pig feed *Salmonella* control was justified already because of public health issues. Most of the saved costs were associated with lower costs incurred due to contaminations in pigs (whether observed at farm or at slaughterhouse) or in their environment. These costs were saved because of reduced prevalence of *Salmonella* in pigs or in their environment and measures associated with these contaminations because of FSCP. Hence, effective pig feed *Salmonella* control can support effectiveness of FSCP, which further supports public health in Finland. Pig feed *Salmonella* control policy and FSCP should therefore be examined jointly. An important aspect which can be generalized beyond the context of this study is that risk management measures can be complementary when applied in a livestock supply chain and the use of joint inputs is involved, and therefore, possible joint effects of risk management measures affecting the same outcome indicators should be considered.

Potential limitations of our study are related to the scenarios which were investigated. Besides the two scenarios, we did not look at other intervention scenarios. Hence, there may be other scenarios with slightly reduced or increased control options which could be preferred over the current control policy. This includes novel combinations where both pig feed *Salmonella* control policy and FSCP are adjusted from their current status. The scenarios were also developed by using information from a limited number of feed operators, which, together with missing responses, causes uncertainty about the parameter values used. Specific conditions prevailing in Finland may limit the applicability of the results to other contexts. Another potential limitation is related to the appraisal of joint costs. This refers to the costs of measures such as biosecurity, which are applied to mitigate several diseases. Accounting for a higher proportion of these costs to be related to *Salmonella* prevention would increase the costs of the current control scenario. However, apart from feed heat treatment, the costs were rather modest.

### Stakeholder Incentives

Strict liability currently defines responsibilities regarding feed-related *Salmonella* contaminations. These liabilities are mainly faced by the commercial feed manufacturers as they must indemnify damage caused by contaminated feed for buyer if their feed does not meet the requirement of the law, even though the contamination is not caused by intent or negligence. This can be a financially heavy responsibility. The reduced control scenario examined a situation where more responsibility on the contamination costs and human cases was transferred to the pig industry and consumer. Since the beneficiaries of strict liability are mainly farms which purchase pig feed, the meat industry, and consumers who face reduced risk of salmonellosis both directly and indirectly through support that it provides to FSCP's goals, an important policy question is whether the feed suppliers are able to recover their costs through feed prices.

The incentive aspect can be extended beyond the context of this study. In order to provide sufficient incentives to comply with a given level of liability, it would be important that feed suppliers can recover their costs, for instance, through an insurance policy, a co-finance mechanism involving the supply chain parties, or from the markets. Even if the official pig feed *Salmonella* control policy would be relaxed, many similar actions, and thus their costs, would be taken as they are part of the policy to control animal diseases and to maintain appropriate feed hygiene. Official control and self-control measures should therefore be considered jointly.

The cost aspect is relevant because previous studies show in the context of farms that there is a clear inverse relationship between the willingness of farmers to adopt a biosecurity measure to reduce *Salmonella* and its estimated cost [Niemi et al. (39), Fraser et al. (40)]. For an individual farm in Finland, the damage caused by *Salmonella* contamination can be substantial. An elevated risk of *Salmonella* can occur when interventions are applied only at later stages of the supply chain. With the statutory requirement to eliminate *Salmonella*, the consequences would be costly for the pig producers. Insurance policies to cover the risk of *Salmonella* contamination exist, but they are sometimes considered expensive. This is a challenge not only in Finland but also in various other countries where livestock disease insurance is available in the market [cf. Heikkilä and Niemi (41)].

## Conclusion

The current pig feed *Salmonella* control policy of Finland is economically profitable because it reduces the costs caused by *Salmonella* contaminations along the food chain with low costs. This provides public health benefits which are higher than the costs of pig feed *Salmonella* control. Pig feed *Salmonella* control can support the effectiveness of a broader control program (FSCP), which further enhances public health. Pig feed *Salmonella* control policy and FSCP should therefore be examined jointly. In order to increase the acceptability of current policy, greater attention to the allocation of financial responsibilities (costs and benefits) regarding the control measures may be required.

## Data Availability

The datasets for this manuscript are not publicly available because the data contain confidential information which cannot be revealed to parties other than scientists who were involved in the study. Requests to access the datasets should be directed to jarkko.niemi@luke.fi.

## Author Contributions

KH carried out a financial analysis on the current control program. KH and MS carried out most of the data collection. PT led the risk assessment work. JN led the economic analysis and conducted stochastic simulations to assess the overall costs and costs caused by *Salmonella* contaminations. JN and KH drafted the manuscript. All authors contributed to the planning of the study and reporting the results.

### Conflict of Interest Statement

The authors declare that the research was conducted in the absence of any commercial or financial relationships that could be construed as a potential conflict of interest.
