# Supplementary material for: Salmonella Control Programme of Pig Feeds Is Financially Beneficial in Finland
Source: Front Vet Sci. 2019 Jul 9;6:200. doi: 10.3389/fvets.2019.00200 (PMC6629760; doi:10.3389/fvets.2019.00200)
Supplement: Supplementary file 1 [file Presentation_1.pdf]

Supplementary material for the article "*Salmonella* control programme of pig feeds is financially beneficial in Finland"

Jarkko K. Niemi[1], Katriina Heinola[2], Maria Simola[3], Pirkko Tuominen[3],

[1] Bioeconomy and Environment, Natural Resources Institute Finland (Luke), Seinäjoki, Finland
[2] Bioeconomy and Environment, Natural Resources Institute Finland (Luke), Helsinki, Finland
[3] Risk Assessment Unit, Finnish Food Authority, Helsinki, Finland

Correspondence: jarkko.niemi@luke.fi

Appendix 1. Parameters describing the prevalence of salmonella feed materials and feed imported into Finland (proportion of 25 ton lots of imported feed contaminated), and the prevalence of salmonella in feed materials and feed manufactured (proportion of 25 ton lots of feed or feed material) in Finland in the current control scenario.

	Mean	sd	2.5 %	97.5 %
**Imported feed or feed materials**				
Sunflower	0.001595	0.002282	0.000002	0.008090
Whey	0.000133	0.000246	0.000000	0.000705
Pea	0.005112	0.007815	0.000006	0.026330
Oats	0.000093	0.000061	0.000015	0.000244
Milk	0.001103	0.001603	0.000001	0.005626
Barley	0.000062	0.000042	0.000010	0.000167
Brewer's yeast	0.001322	0.001886	0.000001	0.006714
Bean	0.001713	0.002470	0.000001	0.008657
Linen seed	0.021370	0.030260	0.000019	0.107400
Rank	0.000174	0.000383	0.000000	0.000929
Rapeseed, domestic origin	0.000239	0.000337	0.000000	0.001204
Rapeseed, imported	0.013670	0.002246	0.009862	0.018690
Soybean	0.003319	0.002150	0.000534	0.008694
Soybean, imported	0.004425	0.001182	0.002465	0.007080
Sugar beet chips	0.000494	0.000709	0.000000	0.002522
Sugar beet chips, imported	0.001764	0.001463	0.000123	0.005555
Wheat	0.000091	0.000060	0.000015	0.000241
Wheat bran	0.006753	0.002395	0.002973	0.012250
Complete feed for sows	0.037750	0.054050	0.000032	0.192800
Complete feed for fattening pigs	0.038370	0.053360	0.000038	0.189900
**Feed materials and feed manufactured in Finland**				
Industrial complete feed for sows	$5.10*10^{-6}$	$7.38*10^{-6}$	$2.01*10^{-7}$	$2.81*10^{-5}$
Industrial complete feed for pigs	$4.23*10^{-6}$	$6.13*10^{-6}$	$1.69*10^{-7}$	$2.32*10^{-5}$
Supplementary feed for sows	$1.13*10^{-5}$	$1.65*10^{-5}$	$4.34*10^{-7}$	$6.24*10^{-5}$
Supplementary feed for fattening pigs	$1.51*10^{-5}$	$2.17*10^{-5}$	$5.91*10^{-7}$	$8.31*10^{-5}$
Supplementary liquid feed	$6.36*10^{-7}$	$2.15*10^{-6}$	$6.36*10^{-9}$	$4.37*10^{-6}$
Feed prepared at the farm for liquid feeding (without supplementary feed)	$9.11*10^{-5}$	$4.54*10^{-5}$	$4.65*10^{-5}$	$1.92*10^{-4}$
Feed prepared at the farm for dry feeding (without supplementary feed)	$6.38*10^{-5}$	$1.87*10^{-5}$	$3.71*10^{-5}$	$1.09*10^{-4}$
Imported complete feed for sows	0.01596	0.02280	$1.37*10^{-5}$	0.08153
Imported complete feed for fattening pigs	0.01625	0.02265	$1.62*10^{-5}$	0.08042

Appendix 2. Parameters describing the prevalence of salmonella contamination in feed fed to pigs (proportion of 25 ton lots of feed contaminated) in the current control scenario[1].

	Mean	sd	2.5 %	97.5 %
Industrial complete feed for sows	0.001013	0.000213	0.000661	0.001488
Industrial complete feed for pigs	0.000842	0.000176	0.000556	0.001243
Liquid on-farm mixture with industrial complementary feeds for sows	0.000320	0.000119	0.000190	0.000576
Liquid on-farm mixture with industrial complementary feeds for pigs	0.000361	0.000120	0.000228	0.000620
Dry on-farm mixture with industrial complementary feeds for sows	0.000426	0.000981	0.000273	0.000644
Dry on-farm mixture with industrial complementary feeds for pigs	0.000524	0.000102	0.000363	0.000752
Liquid on-farm mixture with industrial liquid complementary feed for sows	0.000205	0.000122	0.000099	0.000463
Liquid on-farm mixture with industrial liquid complementary feed for pigs	0.000205	0.000122	0.000099	0.000463
Liquid farm mixture with industrial liquid feed (complementary <10%) for sows	0.000209	0.000116	0.000104	0.000456
Liquid farm mixture with industrial liquid feed (complementary <10%) for pigs	0.000209	0.000012	0.000104	0.000456
Dry on-farm mixture with industrial liquid complementary feed for sows	0.000150	0.000061	0.000083	0.000282
Dry on-farm mixture with industrial liquid complementary feed for pigs	0.000150	0.000061	0.000083	0.000282
Imported complete feed for sows	0.037750	0.054050	0.000032	0.192800
Imported complete feed for pigs	0.038370	0.053360	0.000038	0.189900

[1] Parameter values in the table represent the true prevalence. Based on the data, apparent prevalence was estimated at 42% of the true prevalence. In addition, it was estimated that 11% of cases lead to the culling of the pigs due to *Salmonella* contamination.
